# The mediating pathways of frailty on healthcare costs and length of stay in older patients with colorectal cancer: a multicenter retrospective study

**DOI:** 10.3389/fmed.2026.1854599

**Published:** 2026-07-30

**Authors:** Yixiang Huang, Jianchao Liu, Runda Jiao, Cheng Li, Xiangbo Zhang, Xiaohui Yuan, Jiacheng Xu, Lihua Liu, Tianyi Zhang

**Affiliations:** 1 Graduate School, Chinese PLA General Hospital, Beijing, China; 2 Department of Medical Innovation and Research, Chinese PLA General Hospital, Beijing, China

**Keywords:** CRC, economic burden, elderly patients, frailty, mediation analysis

## Abstract

**Background:**

The relationship between frailty and healthcare resource utilization in older patients with colorectal cancer (CRC) remains insufficiently quantified. We evaluated the associations of frailty with high inpatient costs, length of stay (LOS), and adverse events, and examined the explanatory roles of co-occurring comorbidity burden and postoperative complications.

**Methods:**

This multicenter retrospective study analyzed 4,936 patients aged ≥60 years undergoing elective CRC surgery between 2014 and 2024. Hierarchical multivariable regression models evaluated associations between frailty and outcomes. Mediation-style decomposition was used to estimate the extent to which comorbidity burden and postoperative complications explained frailty-associated differences in high inpatient costs and LOS. A simulation-based sensitivity analysis was performed to assess the potential impact of ICD-10-based frailty misclassification. A Bonferroni-corrected threshold of *p* < 0.006 was applied.

**Results:**

Frailty was significantly associated with high inpatient costs (OR = 1.274, 95% CI: 1.083–1.498, *p* = 0.003), prolonged LOS (log-transformed *β* = 0.082, 95% CI: 0.053–0.111, *p* < 0.001), and adverse events (OR = 3.317, 95% CI: 2.267–4.854, *p* < 0.001). Mediation-style decomposition showed that co-occurring comorbidity burden explained 28.6% of frailty-associated difference in high inpatient costs (*p* < 0.001). Postoperative complications did not significantly explain the frailty–LOS association (3.8%, *p* = 0.368). The estimated excess socioeconomic burden associated with frailty was CNY 657 million. In the sensitivity analysis, the direction of frailty associations with all three outcomes remained unchanged across simulated ICD-10 misclassification scenarios, although the association with high inpatient costs was attenuated under moderate and severe assumptions.

**Conclusion:**

Frailty was associated with increased healthcare resource utilization and adverse outcomes in older CRC patients. Co-occurring comorbidity burden partly explained the frailty-associated difference in high inpatient costs, whereas prolonged LOS was not significantly explained by postoperative complications. These findings support routine frailty screening and dual-track perioperative optimization targeting both comorbidity burden and frailty-related physiological vulnerability.

## Introduction

Frailty is defined as an age-related syndrome of multisystem physiological decline, characterized by diminished reserve and increased vulnerability to stressors, which significantly results in adverse health outcomes and increases healthcare resource utilization (1). Based on China’s “expert consensus” definition, about 10% of people aged 60 and over, 15% of people aged 75–84, and 25% of people aged 85 and over suffer from frailty (2). The clinical correlation between frailty and cancer has been well-established (3). A UK Biobank study revealed that cancer patients with multimorbidity (≥ 2 chronic conditions) had 43% higher odds of pre-frailty/frailty, escalating to 2.03-fold when ≥ 2 additional comorbidities coexisted (4). Frailty and chronic disease are commonly perceived as distinct concepts in the context of chronic disease management. However, these two concepts are interrelated and exhibit a certain degree of overlap, as the occurrence of chronic diseases can lead to frailty, which in turn can alter the risk benefit status of interventions for chronic diseases (5). Despite the established link between frailty and chronic diseases, its specific impact on colorectal cancer-a condition with rising incidence and economic burden in aging populations-remains underexplored, particularly in resource allocation contexts.

Globally, 2.17 million new colorectal cancer (CRC) cases and 926,000 deaths were estimated in 2019, representing increases of 157 and 110%, respectively, since 1990. Incidence peaked at ages 65–69 in men and 70–74 in women, with mortality rising with age. The number of CRC cases is predicted to reach 6.098 × 10^6^ between 2020 and 2044, about three times the number in 2019; meanwhile, the number of global CRC deaths will continue to increase (6). The total economic burden of CRC in China in 2019 is estimated to be RMB 170.5 billion, accounting for 0.173% of China’s gross domestic product (GDP) in that year, with a direct economic burden of RMB 106.4 billion, or about 62.4% of the total economic burden. The economic burden of CRC in China is expected to increase by 228.4% by 2030 compared with 2019 (7). In 2022, the CRC incidence and mortality ratios in China are greater than those in the United States, and the incidence rates of CRC in both men and women in China have been increasing since 2000, whereas the United States have been decreasing substantially (8).

Current frailty assessments predominantly rely on clinician-administered scales. While these tools are validated (9), their reliance on active data collection (e.g., grip strength, questionnaires) introduces time constraints and subjective bias. In contrast, ICD-10-based screening offers a pragmatic alternative for rapid patient stratification, particularly in emergency settings (10).

Most studies analyze only simple associations between frailty and outcomes and ignore mediating effects, resulting in an inability to reveal the specific mechanisms by which frailty affects healthcare resource consumption and contributes to adverse outcomes through critical pathways, limiting the precision of clinical interventions.

This multicenter retrospective study examined the impact of frailty, identified via ICD-10 codes, on healthcare use and adverse outcomes in elderly patients with colorectal cancer. We examined whether co-occurring comorbidity burden and postoperative complications statistically explained frailty-associated healthcare utilization, providing evidence to guide resource allocation and high-risk patient identification.

## Methods

### Study design

The study was a retrospective cohort observational study of patients aged 60 years and older who underwent elective colorectal cancer surgery from 2014 to 2024 at five medical centers affiliated with a comprehensive group hospital, Beijing, a tertiary teaching facility with multiple specialties, including geriatric medicine. Frailty was identified using the ICD-10 diagnostic codes outlined in the validation study by Gilbert et al. (11). A patient was classified as frail if their admission diagnosis included any specific frailty-defining code listed in the appendix of Gilbert’s study (Appendix S1). This study hypothesized that frailty-associated healthcare resource utilization would be partly explained by co-occurring comorbidity burden and postoperative complications, while a residual association with frailty would remain after accounting for these clinical burdens. Because comorbidity burden was measured at or around admission, it was interpreted as a co-occurring contributory burden rather than as a strictly time-ordered causal mediation. In contrast, postoperative complications occurred after surgery and before discharge, making them temporally plausible contributors to LOS, although LOS also included preoperative hospital days. This study was exempt from institutional review board approval because all the data were historically de-identified.

### Data collection

Patient information was extracted from the Hospital Information System (HIS), including demographic characteristics (age and sex), admission-related characteristics (admission status and condition at admission), comorbidity burden, surgical and oncological characteristics, and clinical outcomes. Admission route was used to identify emergency admission, and condition at admission was used to identify admission severity. Tumor stage was categorized as metastatic or non-metastatic disease according to metastasis status. Surgical approach was classified as minimally invasive surgery or open surgery. ICD-10 based frailty assessment was conducted by qualified researchers utilizing retrospectively gathered clinical data derived from pre- and post-operative records documented by physicians, nurses, and allied health professionals. Researchers remained unaware of patient outcomes during the frailty assessment process. Frailty status and comorbidity index were defined using information available at or around admission. Postoperative complications were recorded after surgery during the index hospitalization. LOS was calculated from admission to discharge and therefore included both preoperative and postoperative hospital days. Accordingly, postoperative complications were considered temporally plausible contributors to LOS, although the inclusion of preoperative hospital days was considered when interpreting the mediation-style estimates.

### Statistical analysis

To eliminate interference with costs in different years, all costs from prior years were adjusted to 2024 CNY using CPI (12) (Appendix S2).

For logistic regression analysis, total inpatient costs were dichotomized into a binary outcome. High inpatient costs were defined as total costs exceeding the 75th percentile of the distribution for the entire cohort (CNY 99,318.49). LOS was treated as a continuous outcome. Because LOS was right-skewed, it was natural-log transformed before inclusion in the linear regression models.

Non-normally distributed continuous variables were expressed as median (quartiles), i.e., median (P25, P75), and the Mann–Whitney *U* test was performed for the comparison. Categorical variables were expressed as frequency (constitutive ratio), i.e., *n* (%), and a chi-square test was conducted for the comparison. To investigate the effects of different variables on outcomes and changes in effects, hierarchical logistic regression analyses were used for inpatient costs and the presence of adverse events, and hierarchical linear regressions were used for LOS. Mediation-style decomposition analyses were performed to estimate the extent to which selected clinical burdens statistically explained the associations between frailty and healthcare outcomes. Specifically, the comorbidity index was examined as a co-occurring burden explaining the frailty–cost association, and postoperative complications were examined as a temporally plausible contributor to the frailty–LOS association. Average mediation effect, average direct effect, and mediation proportions were reported. Given the retrospective design and the timing of comorbidity measurement, these estimates were interpreted as explanatory statistical decompositions rather than definitive causal effects. The ADE from the mediation-style decomposition was not interpreted interchangeably with an independent predictor from multivariable regression. The former represents the residual association after accounting for the specified mediator, whereas the latter refers to the association estimated after adjustment for covariates in regression models. We assessed model fit and multicollinearity by verifying calibration with the Hosmer-Lemeshow test (*p* > 0.05), checking multicollinearity using VIF < 5, and testing robustness through stepwise adjustment. All statistical analyses were performed using R version 4.4.2. Statistical significance was initially set at *p* < 0.05. To control for the type I error rate due to multiple comparisons across outcomes and sequential models, a Bonferroni-corrected threshold of *p* < 0.006 was applied to the multivariable analyses.

### Sensitivity analysis

To assess the potential impact of ICD-10-based frailty misclassification, we performed a simulation-based probabilistic bias analysis. Similar simulation-based and uncertainty analyses have been used in recent epidemiological studies to evaluate the robustness of model-based estimates (13). Because ICD-10-based frailty identification is generally characterized by relatively low sensitivity and high specificity, three scenarios were specified: mild misclassification with sensitivity/specificity of 0.70/0.98, moderate misclassification with sensitivity/specificity of 0.60/0.97, and severe misclassification with sensitivity/specificity of 0.50/0.95. For each scenario, latent frailty status was repeatedly simulated according to the assumed sensitivity and specificity, and the fully adjusted models for high inpatient costs, log-transformed LOS, and adverse events were re-estimated across repeated simulations. Results were summarized as median estimates with 2.5th–97.5th percentiles. Statistical significance was assessed using the Bonferroni-corrected threshold of *p* < 0.006.

### Social health economy

A health economic analysis was conducted to estimate the potential socioeconomic burden attributable to frailty and the potential savings from targeted interventions. The full methodology, including the calculation formula and data sources, is detailed in the Appendix S3.

## Results

### Patient characteristics

A total of 4,936 patients were included (3,877 non-frail, 1,059 frail). Baseline characteristics are presented in Table 1. While sex (*p* = 0.621) and tumor stage (*p* = 0.586) distribution was similar, frail patients were significantly older and had higher rates of emergency admission, critical illness, higher costs, longer LOS, higher comorbidity index, unplanned return to the operating room, adverse events, and complications, lower rate of minimally invasive surgery (all *p* < 0.05) (Table 1).

**Table 1:** Characteristics and outcomes of frail and non-frail patients with colorectal cancer.

Characteristics	Total patients (*N* = 4,936)	Non-Frail patients (*N* = 3,877)	Frail patients (*N* = 1,059)	*P*
**Age**	**69.00 (64.00, 74.00)**	**68.00 (64.00, 74.00)**	**71.00 (66.00, 77.00)**	< 0.001
**Sex**				0.621
Male	2,969 (60.15)	2,339 (60.33)	630 (59.49)	
Female	1,967 (39.85)	1,538 (39.67)	429 (40.51)	
**Emergency admission**				< 0.001
YES	686 (13.90)	474 (12.23)	212 (20.02)	
NO	4,250 (86.10)	3,403 (87.77)	847 (79.98)	
**Admission severity**				< 0.001
Critical illness	744 (15.07)	516 (13.31)	228 (21.53)	
Stable condition	4,192 (84.93)	3,361 (86.69)	831 (78.47)	
**Adjusted Inpatient costs (CNY)**	**78,582.56** **(64,975.96, 99,318.49)**	**77,008.71** **(64,036.12, 96,974.30)**	**83,967.47** **(68,657.96, 107,931.20)**	< 0.001
**Length of stay**	**13.00 (10.00, 17.00)**	**13.00 (10.00, 16.00)**	**15.00 (11.00, 21.00)**	< 0.001
**Comorbidity index**	**0.00 (0.00, 14.00)**	**0.00 (0.00, 14.00)**	**4.00 (0.00, 14.00)**	< 0.001
**Unplanned return to the operating room**				< 0.001
Yes	81 (1.64)	47 (1.21)	34 (3.21)	
NO	4,855 (98.36)	3,830 (98.79)	1,025 (96.79)	
**Adverse events**				< 0.001
YES	129 (2.61)	58 (1.50)	71 (6.70)	
NO	4,807 (97.39)	3,819 (98.50)	988 (93.30)	
**Complications**				< 0.001
Yes	46 (0.93)	7 (0.18)	39 (3.68)	
No	4,890 (99.07)	3,870 (99.82)	1,020 (96.32)	
**Tumor stage**				0.586
Metastatic	1,213 (24.57)	946 (24.40)	267 (25.21)	
Non-metastatic	3,723 (75.43)	2,931 (75.60)	792 (74.79)	
**Surgical approach**				0.007
Minimally Invasive Surgery	3,660 (74.15)	2,909 (75.03)	751 (70.92)	
Open Surgery	1,276 (25.85)	968 (24.97)	308 (29.08)	

Continuous variables are presented as median (interquartile range) and were compared using the Mann–Whitney *U* test. Categorical variables are presented as n (%) and were compared using the chi-square test or Fisher’s exact test, as appropriate. Inpatient costs were adjusted to 2024 CNY.

### Association between frailty and inpatient costs

**Figure 1:**
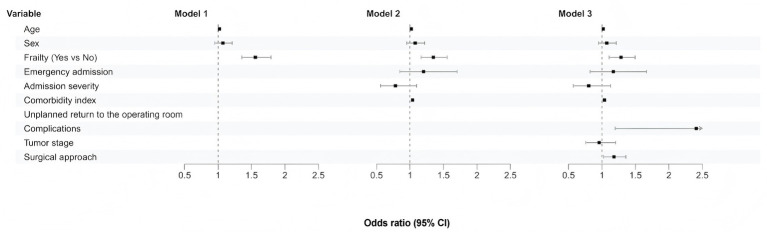
Multivariable logistic regression model for inpatient costs of colorectal cancer patients. Model 1: Adjusted for age, sex, and frailty. Model 2: Adjusted for model 1 plus emergency admission, admission severity, and comorbidity index. Model 3: Adjusted for model 2 plus unplanned return to the operating room, complications, tumor stage, and surgical approach.

Frailty was consistently associated with high inpatient costs across hierarchical models (Figure 1).

In Model 1 (adjusted for age and sex), frail patients demonstrated a 55.7% higher risk of incurring high inpatient costs compared with non-frail patients (OR = 1.557, 95% CI: 1.353–1.791, *p* < 0.001).

In Model 2, after further adjustment for admission characteristics (emergency admission, admission severity) and comorbidity index, the association between frailty and high costs attenuated but remained statistically significant (OR = 1.347, 95% CI: 1.165–1.556, *p* < 0.001). Notably, the comorbidity index itself showed a stable independent effect (OR = 1.038, 95% CI: 1.032–1.045, *p* < 0.001).

In Model 3, which additionally controlled for clinical outcomes (unplanned return to the operating room, complications) and surgical factors (tumor stage, surgical approach), frailty remained significantly associated with high inpatient costs (OR = 1.274, 95% CI: 1.083–1.498, *p* = 0.003).

The attenuation of the frailty estimate after adjustment for comorbidity burden, perioperative clinical events, tumor stage, and surgical approach suggests that part of the frailty–cost association may overlap with these clinical burdens. This observation was further examined using mediation-style decomposition.

### Association between frailty and length of stay

**Figure 2:**
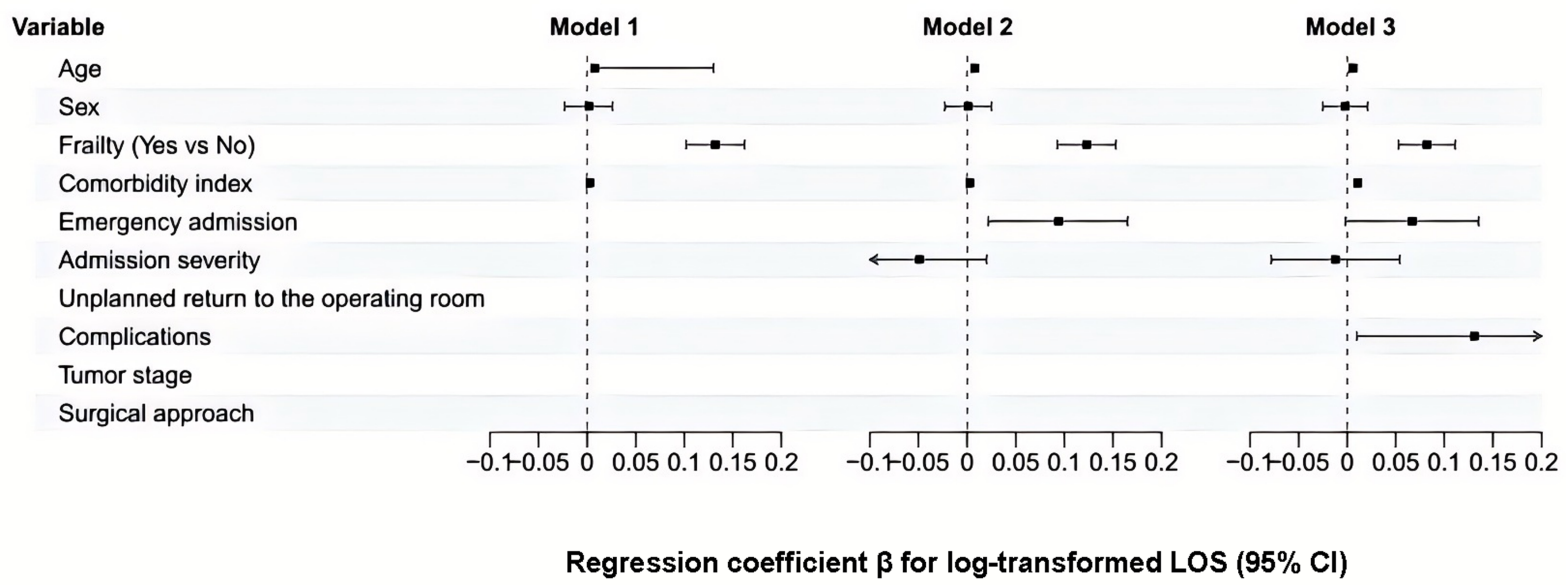
Multiple linear regression model for length of stay of colorectal cancer patients. Model 1: Adjusted for age, sex, frailty, and comorbidity index. Model 2: Adjusted for model 1 plus emergency admission, and admission severity. Model 3: Adjusted for model 2 plus unplanned return to the operating room, complications, tumor stage, and surgical approach.

Multiple linear regression analysis revealed a robust positive association between frailty and prolonged LOS (Figure 2).

In Model 1, frailty was significantly associated with extended hospitalization (*β* = 0.132, 95% CI: 0.102–0.162, *p* < 0.001) after adjusting for demographics.

In Model 2, this association persisted (*β* = 0.123, 95% CI: 0.093–0.153, *p* < 0.001) after controlling for emergency admission and severity.

In the fully adjusted Model 3, despite strong associations observed for unplanned return to the operating room (*β* = 0.541, *p* < 0.001), frailty remained significantly associated with LOS (*β* = 0.082, 95% CI: 0.053–0.111, *p* < 0.001).

The coefficient for frailty decreased from 0.132 in Model 1 to 0.082 in Model 3. However, because the subsequent mediation-style decomposition did not identify a statistically significant indirect component through postoperative complications, this attenuation should be interpreted as overlap with perioperative clinical factors rather than evidence of a confirmed complication-mediated pathway.

### Association between frailty and adverse events

**Figure 3:**
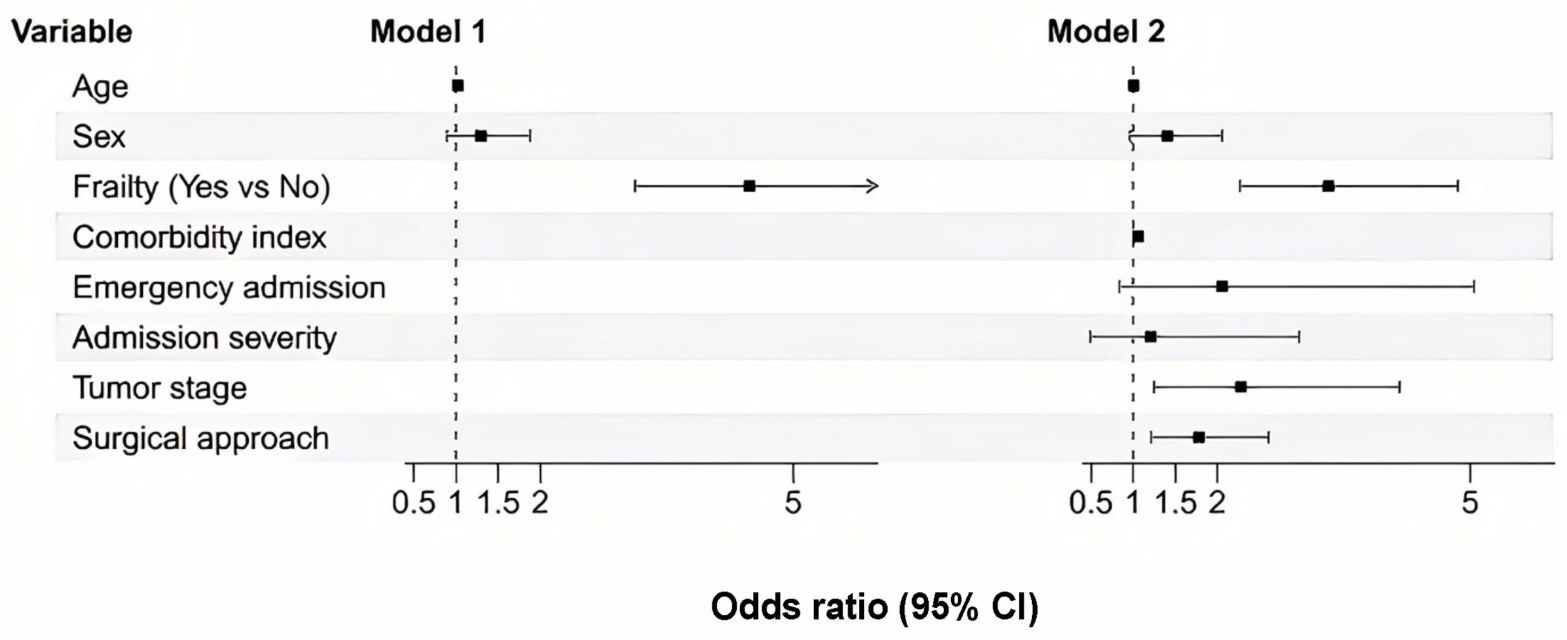
Multivariate logistic regression model for adverse events of colorectal cancer patients. Model 1: Adjusted for age, sex, and frailty. Model 2: Adjusted for model 1 plus emergency admission, admission severity, comorbidity index, tumor stage, and surgical approach.

Logistic regression demonstrated that frailty was strongly associated with postoperative adverse events (Figure 3).

In Model 1, frail patients had a more than four-fold increase in the odds of experiencing adverse events compared to non-frail patients (OR = 4.481, 95% CI: 3.127–6.420, *p* < 0.001).

In Model 2, after adjusting for potential confounders including emergency admission, admission severity, comorbidity index, tumor stage, and surgical approach, frailty remained strongly associated with adverse events (OR = 3.317, 95% CI: 2.267–4.854, *p* < 0.001). The comorbidity index was also significantly associated with adverse events (OR = 1.060, 95% CI: 1.038–1.084, *p* < 0.001).

### The mediation analysis

In the analysis of high inpatient costs, comorbidity burden explained part of the association between frailty and high costs. The total effect estimate was 0.079 (95% CI, 0.045–0.112), with an ADE of 0.057 (95% CI, 0.023–0.091) and an ACME through the comorbidity index of 0.023 (95% CI, 0.015–0.031). The indirect component was statistically significant (*p* < 0.001) and accounted for 28.6% of the total effect. Given that the comorbidity index was measured at or around admission, this result should be interpreted as a mediation-style decomposition through co-occurring comorbidity burden rather than definitive causal mediation.

In the analysis of LOS, frailty was associated with prolonged hospitalization overall (total effect = 0.085, 95% CI: 0.057–0.114). The ADE was 0.082 (95% CI, 0.054–0.111), whereas the ACME through postoperative complications was 0.003 (95% CI, −0.004 to 0.011) and was not statistically significant (*p* = 0.368). Therefore, in this model, postoperative complications coded as a binary composite variable did not significantly explain the frailty–LOS association (Table 2).

**Table 2:** Mediation-style decomposition of frailty-associated healthcare outcomes.

Outcome	Mediator	Total effect (95% CI)	ADE (95% CI)	ACME (95% CI)	% Mediated	*P*
High inpatient costs	comorbidity index	0.079 (0.045–0.112)	0.057 (0.023–0.091)	0.023 (0.015–0.031)	28.6	< 0.001
Length of stay, log-transformed	complications	0.085 (0.057–0.114)	0.082 (0.054–0.111)	0.003 (−0.004–0.011)	3.8	0.368

ADE, average direct effect; ACME, average mediation effect. Estimates are from mediation-style decomposition models. Because the comorbidity index was measured at or around admission, the comorbidity pathway should be interpreted as co-occurring comorbidity burden rather than definitive causal mediation. Postoperative complications were analyzed as a binary composite variable.

### Sensitivity analysis

A simulation-based sensitivity analysis was performed to assess potential ICD-10-based frailty misclassification. Across mild, moderate, and severe misclassification scenarios, the direction of the associations between frailty and all three outcomes remained unchanged. Associations with adverse events and log-transformed LOS remained relatively robust, whereas the association with high inpatient costs was attenuated and did not consistently meet the Bonferroni-corrected threshold under moderate and severe misclassification assumptions (Appendix S4).

### Health economy

According to the latest official government documents, the estimated frailty-associated burdens were CNY 565,448,165.1 for the healthcare system, CNY 45,018,187.95 for households, and CNY 46,897,813.3 for societal productivity. Collectively, the estimated excess socioeconomic burden associated with frailty was CNY 657,364,166.35.

## Discussion

In this large-scale multicenter retrospective study of 4,936 older patients undergoing elective colorectal cancer surgery, the prevalence of ICD-10-defined frailty was 21.4%. Frailty was significantly associated with adverse events, prolonged LOS, and high inpatient costs after adjustment for demographic, admission-related, comorbidity, surgical, and oncological factors. Mediation-style decomposition showed that co-occurring comorbidity burden explained 28.6% of the frailty-associated difference in high inpatient costs, whereas postoperative complications, modeled as a binary composite variable, did not significantly explain the frailty–LOS association. These findings suggest that frailty-related costs are partly linked to concurrent comorbidity burden, while prolonged hospitalization may reflect slower recovery and reduced physiological reserve in frail patients. Given the retrospective design and the timing of comorbidity measurement, these estimates should be interpreted as explanatory statistical decompositions rather than definitive causal pathways.

These findings align with a prospective cohort study (14), which demonstrated that frailty was associated with adverse outcomes, including prolonged LOS, critical illness and death, reinforcing the need for frailty-oriented interventions. Our findings also align with Khanna’s conclusions on the predictive value of the electronic Frailty Index (eFI), but extend their work by examining how comorbidity burden and postoperative complications statistically account for frailty-associated healthcare utilization (15). Notably, even after adjusting for critical prognostic factors such as tumor stage and surgical approach (MIS vs. open), the association between frailty and LOS remained robust. While Nguyen Huy demonstrated the impact of frailty on LOS in cardiac surgery (16), our study refines this understanding in the colorectal cancer setting. In our mediation-style decomposition, postoperative complications did not significantly explain the frailty–LOS association (*p* > 0.05). This does not imply that complications are unimportant for LOS; rather, it suggests that the binary composite complication variable used in this study did not substantially account for prolonged hospitalization among frail patients (17).

In the mediation-style decomposition, the residual association between frailty and LOS was substantially larger than the indirect component through postoperative complications. This finding is consistent with the theory of “impaired stress response” proposed by El Assar (18), suggesting that frail patients may experience slower recovery even in the absence of major recorded complications. Given these distinct explanatory patterns—where high costs are partly linked to co-occurring comorbidity burden and LOS appears more closely related to reduced physiological reserve—a ‘one-size-fits-all’ intervention is insufficient. Therefore, we propose a targeted ‘Dual-Strategy Framework’ to translate these findings into practice. First, to address the cost burden related to comorbidities, systematic preoperative optimization of chronic conditions (e.g., diabetes management, cardiovascular stabilization) through multidisciplinary review is essential to reduce resource consumption (19–21). Second, LOS prolongation in frail patients requires interventions that go beyond simple complication prevention. For elective cases, multimodal prehabilitation programs, including nutrition, exercise, and psychological support, may be initiated 2–4 weeks before surgery to augment physiological reserve and support postoperative recovery. For patients requiring earlier surgery because of urgent symptoms or oncological constraints, abbreviated in-hospital optimization focusing on nutrition, early mobilization, medication review, and comorbidity stabilization may be more feasible (22). In practice, ICD-10-based frailty screening could be performed at admission or surgical scheduling as an electronic trigger for further assessment. Patients identified as frail should then be reviewed by a multidisciplinary team involving colorectal surgery, anesthesiology, geriatrics, nursing, nutrition, and rehabilitation. However, ICD-10-based screening should be regarded as an electronic trigger rather than a stand-alone diagnostic tool, and its implementation in lower-level or underdeveloped hospitals may be limited by coding quality, documentation completeness, geriatric expertise, and electronic medical record infrastructure (23).

Frailty-oriented interventions may reduce LOS at a lower marginal cost (CNY 8,397/patient) than complication-driven care (CNY 11,828/patient) (24, 25). Focusing on frailty status and physiological reserve, rather than solely on postoperative complications, may therefore offer a more cost-effective strategy for reducing prolonged hospitalization. In this study, the estimated excess socioeconomic burden associated with frailty was CNY 657 million. This estimate should be interpreted as an approximate scenario-based burden estimate rather than directly recoverable savings. Nevertheless, it highlights the potential scale of frailty-associated resource use and supports targeted investment in routine frailty screening, comorbidity optimization, and prehabilitation programs (26). This “savings-to-prevention” concept is consistent with international recommendations on applying cost-effectiveness analysis to healthcare resource prioritization (27).

Several limitations should be acknowledged. First, all patients were from tertiary hospitals in Beijing, which may limit generalizability to lower-level, rural, or resource-constrained settings. Differences in coding quality, electronic medical record infrastructure, geriatric expertise, and perioperative care pathways may affect both ICD-10-based frailty identification and healthcare resource use. Secondly, ICD-10-based frailty identification has limited sensitivity and may under-detect mild frailty, leading to contamination of the non-frail reference group. This non-differential misclassification would generally bias the main frailty–outcome associations toward the null, suggesting conservative estimates. Our simulation-based sensitivity analysis showed that the direction of associations remained unchanged, although the association with high inpatient costs was attenuated under moderate and severe misclassification assumptions. However, the influence of misclassification on mediation-style estimates is less certain, because it may either attenuate differences in comorbidity burden between frail and non-frail patients or enrich the observed frail group with more severe multimorbidity. Therefore, mediation proportions should be interpreted cautiously rather than as precise causal quantities. Third, the retrospective administrative dataset lacked long-term follow-up and did not consistently include ASA physical status, nutritional status, functional measures, laboratory markers, or social support. These unmeasured factors may confound the association between frailty and healthcare utilization. Nevertheless, adjustment for tumor stage and surgical approach, which reflect oncological severity and operative complexity, partly mitigated this concern. The persistence of frailty associations after these adjustments suggests that the findings are unlikely to be fully explained by unmeasured case complexity. Fourth, postoperative complications were analyzed as a binary composite variable, which may obscure differences among complications with distinct impacts on LOS, such as anastomotic leakage, pneumonia, ileus, bleeding, or wound infection. Therefore, the non-significant indirect component through overall complications should not be interpreted as evidence that individual complications do not prolong hospitalization. Finally, the absence of a standardized frailty definition across studies may affect cross-study comparability.

Future multicenter prospective studies incorporating standardized frailty assessment tools and long-term follow-up are required to validate these findings and evaluate the impact of frailty on survival, quality of life, and post-discharge resource use (28). Geriatric-surgical co-management models should also be tested to determine whether structured comorbidity optimization and prehabilitation can reduce frailty-associated healthcare utilization (29). Future trials should distinguish complication subtypes and severity, separate preoperative and postoperative LOS, and include cost-effectiveness analyses. In addition, future tumor research may integrate frailty phenotyping with tumor-related molecular or inflammatory biomarkers, as prognostic biomarker models have shown value in refining risk stratification in cancer populations (30). Digital health technologies, such as wearable sensors and machine learning models based on electronic health records, may further improve functional monitoring and frailty phenotyping (31).

## Conclusion

In conclusion, frailty was significantly associated with high inpatient costs, prolonged LOS, and adverse events in older patients undergoing colorectal cancer surgery. Mediation-style decomposition suggested that co-occurring comorbidity burden partly explained the frailty-associated difference in high inpatient costs, whereas postoperative complications did not significantly explain the frailty–LOS association. These findings support a shift from reactive complication management alone toward proactive perioperative optimization. Integrating routine frailty screening into preoperative care may help identify patients who require comorbidity management, physiological reserve enhancement, and targeted rehabilitation. Future efforts should translate these findings into practical dual-track care models addressing both comorbidity burden and frailty-related vulnerability.

## Data Availability

The datasets generated and/or analyzed during the current study are available from the corresponding author upon reasonable request, subject to institutional approval.
