# Supplementary material for: The mediating pathways of frailty on healthcare costs and length of stay in older patients with colorectal cancer: a multicenter retrospective study
Source: Front Med (Lausanne). 2026 Jul 30;13:1854599. doi: 10.3389/fmed.2026.1854599 (PMC13467821; doi:10.3389/fmed.2026.1854599)
Supplement: Supplementary file 1 [file Supplementary_file_1.DOC]

**Supplementary Material**

**Contents**

**Appendix S1. ICD-10 codes relevant to frailty.**

**Appendix S2. Standardized Consumer Price Index (%), 2014-2024.**

**Appendix S3. Supplementary Methods.**

**Appendix S4. Simulation-based sensitivity analysis for potential ICD-10-based frailty misclassification.**

**Table S1 ICD-10 codes relevant to frailty**

The selection of the following ICD-10 codes (Table S1) is strictly based on the validation study by Gilbert et al. published in *The Lancet*.(1) In their study, these codes were identified through cluster analysis of 22,139 older patients and subsequently validated in a national cohort of over 1 million patients. This coding set has demonstrated robust predictive validity for adverse outcomes (including 30-day mortality, long hospital stays, and readmissions) and moderate concordance with the Fried Phenotype and Rockwood Frailty Index, confirming its utility for frailty case-finding in administrative datasets.

**Identification Rule:** A patient was classified as frail if any of the following ICD-10 codes (Table S1) were documented in their admission records.

**Table S1 ICD-10 codes relevant to frailty**

| **ICD-10 code** | **ICD Description** |
| --- | --- |
| **E46** | Unspecified protein-energy malnutrition |
| **F00, F01, F02, F03,F05** | F00* Dementia in Alzheimer's disease |
|  | F00.0* Dementia in Alzheimer's disease with early onset (G30.0+) |
|  | F00.1* Dementia in Alzheimer's disease with late onset |
|  | F00.2* Dementia in Alzheimer's disease, atypical or mixed type |
|  | F00.9* Dementia in Alzheimer's disease, unspecified |
|  | F01 Vascular dementia |
|  | F01.0 Vascular dementia of acute onset |
|  | F01.1 Multi-infarct dementia |
|  | F01.2 Subcortical vascular dementia |
|  | F01.3 Mixed cortical and subcortical vascular dementia |
|  | F01.8 Other vascular dementia |
|  | F01.9 Vascular dementia, unspecified |
|  | F02* Dementia in other diseases classified elsewhere |
|  | F02.0* Dementia in Pick's disease A |
|  | F02.1* Dementia in Creutzfeldt-Jakob disease |
|  | F02.2* Dementia in Huntington's disease |
|  | F02.3* Dementia in Parkinson's disease |
|  | F02.4* Dementia in human immunodeficiency virus [HIV] disease |
|  | F02.8* Dementia in other specified diseases classified elsewhere |
|  | F03 Unspecified dementia |
|  | F05 Delirium, not induced by alcohol and other psychoactive substances Excludes: delirium tremens, alcohol-induced or unspecified ( F10.4 ) |
|  | F05.0 Delirium not superimposed on dementia, so described |
|  | F05.1 Delirium superimposed on dementia |
|  | F05.8 Other delirium |
|  | F05.9 Delirium, unspecified |
| **R15** | Faecal incontinence |
| **R26.2 & R26.8** | R26.2 Difficulty in walking, not elsewhere classified |
|  | R26.8 Other and unspecified abnormalities of gait and mobility |
| **R32** | Unspecified urinary incontinence |
| **R40** | Somnolence, stupor and coma |
|  | Excludes: coma: |
|  | diabetic |
|  | hepatic |
|  | hypoglycaemic (nondiabetic) |
|  | neonatal |
|  | uraemic |
| **R41** | Other symptoms and signs involving cognitive functions and awareness |
|  | R41.0 Disorientation, unspecified |
|  | R41.1 Anterograde amnesia |
|  | R41.2 Retrograde amnesia |
|  | R41.3 Other amnesia |
|  | R41.8 Other and unspecified symptoms and signs |
| **R46.0** | Very low level of personal hygiene |
| **R54** | Senility |
| **W00-W19** | Falls |
| **Z73.9** | Problem related to life-management difficulty, unspecified |
| **Z74** | Problems related to care-provider dependency |
|  | Z74.0 Reduced mobility, bedfast, chairfast |
|  | Z74.1 Need for assistance with personal care |
|  | Z74.2 Need for assistance at home and no other household member able to render care |
|  | Z74.3 Need for continuous supervision |
|  | Z74.8 Other problems related to care-provider dependency |
|  | Z74.9 Problem related to care-provider dependency, unspecified |
| **Z99.3** | Dependence on wheelchair |

**Consumer Price Index (CPI) Adjustment**

Refer to Table S2 for the standardized variations in the CPI between 2014 and 2024. The CPI values for each year are sourced from the official data released by the National Bureau of Statistics of China (NBS), and the fixed-base ratio is determined based on the CPI value of the preceding year.(2)

**Table S2 Standardized Consumer Price** Index (%), 2014-2024

| Year | Fixed-base ratio  （2024=100） |
| --- | --- |
| 2014 | 85.53 |
| 2015 | 86.73 |
| 2016 | 88.47 |
| 2017 | 89.89 |
| 2018 | 91.76 |
| 2019 | 94.42 |
| 2020 | 96.78 |
| 2021 | 97.65 |
| 2022 | 99.60 |
| 2023 | 99.80 |
| 2024 | 100.00 |

**Appendix S3: Supplementary Methods**

**Social health economy**

The socioeconomic burden is categorized into three dimensions: healthcare system burden, household burden, and societal productivity burden. This framework adheres to the globally recognized gold standard for societal-perspective cost-effectiveness analysis (CEA) as established by the *Second Panel on Cost-Effectiveness in Health and Medicine*, spearheaded by the US National Academy of Medicine (NAM).(3) This standard ensures the comprehensiveness and scientific rigor of societal cost accounting. All underlying data were sourced from the latest official government documents. Monetary parameters were adjusted to 2024 values using the Consumer Price Index (CPI). The specific calculations employed the following formula:

**Appendix S4. Simulation-based sensitivity analysis for potential ICD-10-based frailty misclassification.**

| **Outcome** | **Scenario** | **Sensitivity** | **Specificity** | **Estimate (interval)** | **Median P value** | **Proportion P < 0.006** |
| --- | --- | --- | --- | --- | --- | --- |
| High inpatient costs, OR | Observed ICD-10 frailty | - | - | 1.274 (1.083-1.498) | 0.003 | 1.000 |
|  | Mild misclassification | 0.70 | 0.98 | 1.177 (1.069-1.295) | 0.031 | 0.202 |
|  | Moderate misclassification | 0.60 | 0.97 | 1.144 (1.025-1.279) | 0.065 | 0.125 |
|  | Severe misclassification | 0.50 | 0.95 | 1.111 (0.985-1.255) | 0.137 | 0.067 |
| Length of stay (log-transformed), β | Observed ICD-10 frailty | - | - | 0.082 (0.053-0.111) | <0.001 | 1.000 |
|  | Mild misclassification | 0.70 | 0.98 | 0.053 (0.037-0.071) | <0.001 | 0.976 |
|  | Moderate misclassification | 0.60 | 0.97 | 0.044 (0.026-0.062) | <0.001 | 0.848 |
|  | Severe misclassification | 0.50 | 0.95 | 0.034 (0.015-0.053) | 0.006 | 0.512 |
| Adverse events, OR | Observed ICD-10 frailty | - | - | 3.317 (2.267-4.854) | <0.001 | 1.000 |
|  | Mild misclassification | 0.70 | 0.98 | 2.462 (1.976-3.059) | <0.001 | 1.000 |
|  | Moderate misclassification | 0.60 | 0.97 | 2.227 (1.731-2.863) | <0.001 | 0.988 |
|  | Severe misclassification | 0.50 | 0.95 | 1.911 (1.427-2.546) | <0.001 | 0.840 |

Values for the observed ICD-10 frailty model are reported as point estimates with 95% confidence intervals. Values for the simulated misclassification scenarios are reported as median estimates with 2.5th–97.5th percentiles across repeated simulations. Mild, moderate, and severe misclassification scenarios assumed ICD-10 frailty sensitivity/specificity of 0.70/0.98, 0.60/0.97, and 0.50/0.95, respectively. OR indicates odds ratio; beta indicates regression coefficient for log-transformed length of stay. Statistical significance was assessed using the Bonferroni-corrected threshold of P < 0.006.

**References**

1.	Gilbert T, Neuburger J, Kraindler J, Keeble E, Smith P, Ariti C, et al. Development and validation of a Hospital Frailty Risk Score focusing on older people in acute care settings using electronic hospital records: an observational study. Lancet. 2018;391(10132):1775-82.

2.	Chen C, Zhang M, Tang J, Pu K. Cost-effectiveness of robotic surgery compared to conventional laparoscopy for the management of early-stage cervical cancer: a model-based economic evaluation in China. BMJ Open. 2024;14(11):e087113.

3.	Sanders GD, Neumann PJ, Basu A, Brock DW, Feeny D, Krahn M, et al. Recommendations for Conduct, Methodological Practices, and Reporting of Cost-effectiveness Analyses: Second Panel on Cost-Effectiveness in Health and Medicine. Jama. 2016;316(10):1093-103.
